# Immunogenicity in mice and rhesus monkeys vaccinated with recombinant vaccinia virus expressing bivalent E7E6 fusion proteins from human papillomavirus types 16 and 18

**DOI:** 10.1186/1743-422X-8-302

**Published:** 2011-06-15

**Authors:** Li Zhao, Binlei Liu, Jiao Ren, Jing Feng, Zheng Pang, Jian Gao, Hui Zhang, Wenjie Tan, Houwen Tian, Li Ruan

**Affiliations:** 1 State Key Laboratory for Molecular Virology and Genetic Engineering, Biotech Center for Viral Disease Emergency, National Institute for Viral Disease Control and Prevention, China CDC, No.155 Changbailu, Changpingqu, Beijing, 102206, China; 2 Department of Immunology, Cancer Institute and Hospital, Chinese Academy of Medical Sciences, 17 Panjiayuan Nanli, Chaoyang District, Beijing, 100021, China

## Abstract

**Background:**

Persistent infection with high-risk human papillomavirus (HPV) is a predominant cause of cervical cancer, and HPV16 and HPV18 occur in 50% and 20% of cervical cancer cases, respectively. The viral oncogenes E6 and E7 are constitutively expressed by HPV-associated tumour cells and can therefore be used as target antigens for immunotherapy. In this study, we constructed a recombinant vaccinia virus co-expressing the HPV16/18 E7E6 fusion proteins (rVVJ16/18E7E6) for use as a therapeutic vaccine for the treatment of HPV16^+ ^and HPV18^+ ^cancers.

**Methods:**

We constructed a bivalent recombinant vaccinia virus expressing modified E7E6 fusion proteins of HPV type 16 and 18 (rVVJ16/18E7E6) based on the vaccinia virus Tiantan strain. We then defined the cellular immune responses to the virus in mice and rhesus monkeys and assessed antitumour efficacy of these responses in mice using the TC-1 tumour challenge model.

**Results:**

Our data demonstrated that rVVJ16/18E7E6 was able to elicit varying levels of CD8^+ ^T cell immune responses and lysis of target cells in mice in response to peptides HPV16E7_49-57 _and HPV18E6_67-75_. Furthermore, the virus was also able to induce anti-tumour responses in the HPV16^+ ^TC-1 tumour challenge model, including partial protection (30-40%) and delayed tumour appearance. In addition, the virus was able to induce immune responses in rhesus monkeys.

**Conclusions:**

The recombinant vaccinia virus rVVJ16/18E7E6 can generate clear and significant cellular immunity in both mice and rhesus monkeys. These data provide a basis for the use of this recombinant virus as a potential vaccine candidate for further study.

## 1. Background

Cervical cancer is the second most common cause of cancer death in women worldwide [1]. Infection with HPV can be demonstrated in 99.7% of cervical cancer patients [2]. Among the high-risk HPV types isolated from cervical carcinomas, HPV16 is the most prevalent, occurring in 46-63% of squamous cell carcinomas, and HPV18 causes about 37-41% of cervical adenocarcinomas worldwide [3]. Two prophylactic HPV vaccines (Gardasil and Cervarix) [4] have been shown to prevent most high-risk HPV infections and to minimise the consequences of HPV-associated diseases. However, these prophylactic vaccines are not predicted to be available in the near future in developing countries due to economic restrictions. In addition, these vaccines have been shown to be effective only in adolescents with no history of previous HPV infection and have not shown a therapeutic effect against current HPV infection or associated lesions [4]. Therefore, a large population will remain at risk of HPV infection in the years to come. For these reasons, the development of a therapeutic vaccine against high-risk HPV is important.

Various immunotherapeutic strategies have been shown to be able to elicit strong immune responses that can eliminate infected cells and lead to tumour regression. The oncoproteins E6 and E7 are constitutively expressed in tumour cells, and their expression is necessary for the transformation and maintenance of the malignant phenotype of the cell [5-7]. Therefore, these viral proteins are used as target antigens for immunotherapy to treat cervical cancer and its precursor intraepithelial lesions. Most studies have focused on therapeutic vaccines against HPV type 16, and, therefore, the E7 and E6 immunodominant epitopes for HPV type 16 and the associated immune responses have been well characterised [8-10]. However, for HPV type 18, another prevalent high-risk type that has been implicated in rapidly developing and potentially aggressive cervical carcinomas [11], there are limited data on therapeutic vaccines.

The vaccinia virus has been accepted as safe, as it was used during the WHO smallpox eradication program. The vaccinia virus Tiantan strain was used as a vaccine against smallpox in China before 1980, and it is now widely used and well tolerated as a vector [12,13]. It has been shown that the Tiantan strain is less virulent when compared with the pathogenic WR strain [14]. In addition, the vaccinia virus induces a strong immune response itself [15]. We previously constructed two recombinant vaccinia viruses expressing modified E6 and E7 fusion proteins from HPV16 and 18, respectively, using the vaccinia virus Tiantan strain [16]. Our previous study indicated that these fusion proteins were able to elicit significant cellular immune responses in mice. Ideally, a potential vaccine candidate should protect against as many HPV types as possible so that it can be used in different patient populations, leading to cost savings in vaccine production and subsequent clinical application. To accomplish this goal, we have constructed a bivalent recombinant vaccinia virus expressing modified E7E6 fusion proteins from both HPV types 16 and 18 (rVVJ16/18E7E6) using the vaccinia virus Tiantan strain. It is important to test whether this recombinant candidate vaccine virus can elicit anti-tumour cellular immune responses in primates. Therefore, we assessed the immunogenicity of rVVJ16/18E7E6 not only in mice but also in rhesus monkeys. These data demonstrate that rVVJ16/18E7E6 is able to elicit specific mouse CTL responses to peptides HPV16E7_49-57 _and HPV18E6_67-75 _and to induce an antitumour response in the HPV16^+ ^TC-1 tumour challenge model. Furthermore, rVVJ16/18E7E6 was able to induce a detectable cellular immune response in rhesus monkeys.

## 2. Materials and methods

### 2.1. Cells and virus

TC-1 cells, generated by co-transformation of primary C57BL/6 mouse lung epithelial cells with HPV-16 E6 and E7 and an activated *ras *oncogene [17], were kindly provided by Dr. T. C. Wu (Johns Hopkins Medical Institutions, Baltimore, Maryland, USA). Primary chicken embryonic fibroblasts (CEF) cells were isolated from 7-8 day-old chicken embryos under sterile conditions. The embryos with head and viscera removed were dissected and washed 3 times with Hank's buffer and then digested with 0.25% trypsin in Hank's buffer at 37°C for 30 minutes. The cell pellets were broken apart by pipetting up and down in minimal essential medium (MEM). The cells were collected by passing them through a filter funnel with 8 gauze layers and resuspended in MEM medium. TC-1 cells and CEF cells were grown in RPMI 1640 medium or MEM medium supplemented with 10% foetal calf serum, 2 mM glutamine, 1 mM sodium bicarbonate, and 100 μg/ml penicillin-streptomycin. Cells were maintained in humidified air containing 5% CO_2 _at 37°C.

Virus rVVJ1175 was a modified Tiantan strain vaccinia virus expressing the LacZ protein in the thymidine kinase (TK) region [18]. The recombinant vaccinia virus rVVJ16E6E7 expressing the E6E7 fusion protein for HPV16 and rVVJ18E7E6 expressing the E7E6 fusion protein for HPV18 were constructed in our lab [16].

### 2.2. Synthetic peptides

Peptide pools consisting of 15 amino acid (AA)-length peptides overlapping by 4 AA and spanning the entire sequence of HPV16E6 (158 AA) and HPV18E7 (90 AA), the D^b ^binding peptide HPV16E7_49-57 _(RAHYNIVTF) [19], and the peptide from HPV18E_67-75 _(KCIDFYSRI) were commercially synthesised (Beijing Scilight Biotechnology Ltd. Co., China). The peptide HPV18E_67-75 _was selected as the optimal peptide for responses in C57BL/6 mice by mapping HPV18E7 peptide pools in a number of pilot experiments carried out in our lab [16]. All peptides were dissolved in DMSO at 50 mg/ml and used at 5 μg/ml in experiments. PMA (50 ng/ml) and ionomycin (1 μg/ml) stimulation was used as a positive control for the generation and detection of antigen-specific T cells by enzyme-linked immunospot assay (Elispot).

### 2.3 Construction of the bivalent recombinant vaccinia virus

#### 2.3.1 Mutagenesis of E7 and E6 for HPV16 and HPV18

The E7 and E6 genes of both HPV types were modified to inactivate their oncogenic activity and then were fused in one reading frame. The E7 protein binds Rb via an L-X-C-X-E motif [7,20]. Therefore, the amino acids at positions ^24^C and ^26^E in HPV16E7 and ^27^C and ^29^E in HPV18E7 were mutated to abolish Rb binding and degradation [21]. For the HPV16E6 protein, mutation at ^57^L or ^63^C has been shown to destroy the HPV16E6 ability to degrade p53 and immortalise primary epithelial cells [6,22,23]. Thus, ^57^L in HPV16E6 and ^65^C in HPV18E6 were altered to inactivate their oncogenic activity. For all mutations, wild type amino acids were mutated to glycine. In addition, the HPV18 E6 coding sequence was mutated to eliminate a potential termination signal (T5NT) for early vaccinia virus transcription without altering the amino acid coding sequence.

The modified HPV16E7E6 fusion gene was amplified with mutagenic primers using overlap extension PCR. Briefly, the mutated version of E7 with the stop codon removed and the mutated version of E6 with the start codon deleted were amplified from pUCmE7-1 [24] and pUCmE6 [24] plasmids, respectively, by using complementary forward and reverse mutagenic primers. The complete E7E6 fusion gene was generated by annealing the two primary PCR products followed by further rounds of PCR with a set of flanking primers. The primary PCR products for the modified E7 and E6 fragments were generated using the following primer pairs: 16E7F (5'-CGGAAGATCTACCATGCATGGAGATACACC-3') with 16E7E6R (5'-CATTGCAGTTCTCTTTTGGTGTGGTTTCTGAGAACAGATGG-3') and 16E7E6F (5'-CCATCTGTTCTCAGAAACCACACCAAAAGAGAACTGCAATG-3') with 16E6R (5'-CTGCAGTTACAGCTGGGTTTC-3'). The complete E7E6 fusion fragment was generated by annealing the two primary PCR products followed by further rounds of PCR with primers 16E7F and 16E6R. The resulting PCR product was cloned into the pMD18-T vector (Takara Dalian, China), resulting in pMD18HPV16E7E6, from which the HPV16E7E6 gene was verified by sequence analysis. The modified HPV18E7E6 fusion gene was commercially synthesised and cloned into the pUC57-T vector (Shanghai Sangon Biological Engineering Technology & Services Co., Ltd. China) to create pUC57HPV18E7E6.

#### 2.3.2 Construction of recombinant vaccinia virus

**Figure 1:**
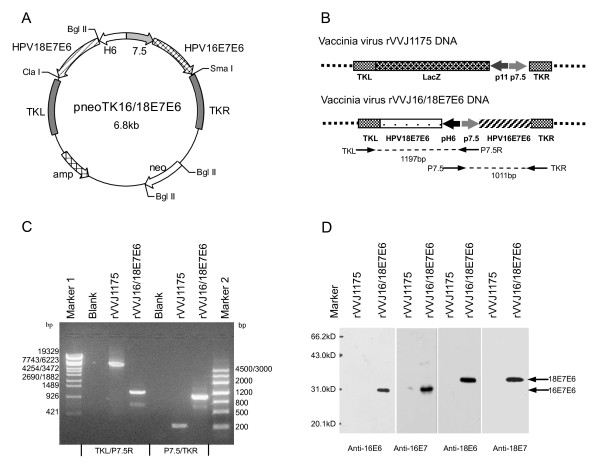
**Construction of recombinant vaccinia virus rVVJ16/18E7E6 and confirmation of the fusion genes by PCR and western blot analyses**. A Schematic diagram of plasmid pneoTKJ16/18E7E6, which contains two expression cassettes in a back-to-back orientation, flanked by vaccinia virus TK region sequences. The expression cassette on the left consists of HPV18E7E6 under the control of the H6 promoter, and the expression cassette on the right is comprised of HPV16E7E6 led by the 7.5 K promoter. Both H6 and 7.5 K promoters are vaccinia-specific promoters. B Schematic diagram of recombinant vaccinia virus rVVJ1175 (top) and rVVJ16/18E7E6 (bottom). Virus rVVJ1175 was a Tiantan strain vaccinia virus with a lacZ gene led by a p11 promoter inserted into the TK region. Virus rVVJ16/18E7E6 was derived from virus rVVJ1175, in which the lacZ expression cassette was replaced by the double expression cassettes for HPV16 and 18 E7E6 fusion proteins. C PCR analyses of the HPV sequences in CEFs infected with rVVJ1175 or rVVJ16/18E7E6 using the primers TKL and P7.5 R and the primers P7.5 and TKR. D Western blot analyses to detect the expression of HPV16E7E6 and HPV18E7E6 fusion proteins in CEFs infected with rVVJ1175 or rVVJ16/18E7E6 using the specific antibodies against HPV16E6, HPV16E7, HPV18E6, or HPV18E7. The marker shown is the low molecular weight protein marker (Institute of Biochemistry and Cell Biology Shanghai Institute for Biological Sciences, China).

The parent virus used for the construction of the recombinant vaccinia virus (rVVJ16/18E7E6) was the rVVJ1175 virus [18]. The plasmid pneoTK16/18E7E6 was constructed and contained the fused HPV16 and HPV18 E7E6 proteins under the control of the vaccinia-specific promoters 7.5 K and H6. The double expression cassettes in pneoTK16/18E7E6 were oriented back-to-back. The plasmids pJSD and pneoTK were used to construct pneoTK16/18E7E6. Both pJSD and pneoTK, described in our previous publications [25,26], contained the vaccinia virus 7.5 K and H6 promoters and flanking sequences spanning the chosen insertion site in the TK region of the J fragment within the vaccinia virus genome. In order to construct pneoTK16/18E7E6, HPV16E7E6 was excised from the plasmid pMD18HPV16E7E6 by digestion with *Bgl *II and *Sma *I and cloned into the *BamH *I and *Sma *I sites of plasmid pJSD under the control of the vaccinia specific 7.5 K promoter, giving rise to pJSDHPV16E7E6. Subsequently, HPV18E7E6 was excised from pUC57HPV18E7E6 by double digestion with *Bgl *II and *Cla *I and inserted into pJSDHPV16E7E6 downstream of the H6 promoter at the *Bgl *II and *Cla *I sites to generate pJSDHPV16/18E7E6. Finally, the double expression cassettes were excised from pJSDHPV16/18E7E6 by *Sma *I and *Cla *I and cloned into pneoTK at the *Sma *I and *Cla *I to create pneoTK16/18E7E6 (as shown in Figure 1A). The final virus vector (rVVJ16/18E7E6) expressing HPV16 and 18 E7E6 fusion proteins was constructed through homologous recombination using rVVJ1175 viral DNA and the plasmid pneoTK16/18E7E6 in CEF cells. The rVVJ16/18E7E6 virus was then purified and propagated.

#### 2.3.3 Polymerase chain reaction (PCR)

CEF cells were infected with the recombinant vaccinia virus rVVJ16/18E7E6 or control virus rVVJ1175 at a multiplicity of infection (MOI) = 1 and incubated for 24 hours. The infected cells were then harvested. Viral DNA was extracted using the DNeasy Blood & Tissue Kit (Qiagen, Germany). Subsequently, PCR (30 cycles of 94°C for 40 seconds, 58°C for 30 seconds and 72°C for 90 seconds) was performed using two pairs of primers containing the promoter sequence and the bilateral sequence of the recombinant vaccinia viruses (P7.5 for 5'-CACTAATTCCAAACCCACCC-3'; P7.5 R for 5'-GGGTGGGTTTGGAATTAGTG-3'; TKR for 5'-CCATCGAGTGCGGCTAC-3'; TKL for 5'-CTATGTGATGTCTTGGAATC-3'). All amplified DNA fragments were sequenced to confirm that the rVVJ16/18E7E6 contained the correct target gene.

#### 2.3.4 Western blotting

CEF cells were infected with the recombinant vaccinia virus rVVJ16/18E7E6 or control virus rVVJ1175 at MOI = 10 and incubated for 24 hours. The infected cells were then harvested, washed once with PBS and resuspended in protein loading buffer (50 mM Tris-Cl pH 6.8, 2% SDS, 10% glycerol, 5% β-mercaptoethanol and 0.025% Bromophenol blue). The cellular extracts were separated by 12% sodium dodecyl sulphate polyacrylamide gel electrophoresis (SDS-PAGE), electrophoretically transferred onto nitrocellulose membranes (Amersham, UK) and blocked in PBST/5% skim milk. After blocking, the membranes were incubated overnight at 4°C with mouse anti-HPV16 E7 monoclonal antibody (prepared in-house) and other commercially available antibodies (goat anti-HPV16 E6, goat anti-HPV18 E6 or goat anti-HPV18 E7 polyclonal antibodies, Santa Cruz, USA). After five washes with PBST, the membranes were incubated with anti-mouse or anti-goat immunoglobulin G-horseradish peroxidase (IgG-HRP, Sigma, USA) for 2 hours at 4°C and washed again with PBST. All antibodies were diluted in PBST/5% skim milk. The membranes were then detected using a chemiluminescence ECL kit (Pierce, USA).

### 2.4 Mouse studies

#### 2.4.1 Mice

Specific pathogen-free female C57BL/6 aged 6-8 weeks old were purchased from the Institute of Laboratory Animal Sciences, Chinese Academy of Medical Sciences & Peking Union Medical College (CAMS & PUMC) and maintained under pathogen-free conditions at the animal facilities of Institute of Materia Medica, CAMS & PUMC.

#### 2.4.2 Mouse IFN-γ Elispot assay

Groups of C57BL/6 mice (five animals per group) were immunised intraperitoneally with 1 × 10^7 ^pfu recombinant vaccinia virus rVVJ16/18E7E6, virus rVVJ16E6E7, virus rVVJ18E7E6, virus rVVJ1175 or PBS. Mice were boosted once with the same dose two weeks later. Splenocytes were harvested for the analysis of HPV-specific cellular immune responses by Elispot and for intracellular cytokine staining ten or twelve days after the last vaccination.

The mouse IFN-γ Elispot assay was carried out according to the manufacturer's instructions (U-CyTech, Netherlands). Briefly, wells of PVDF membrane-bottomed plates (Millipore, Netherlands) were coated with the anti-mouse IFN-γ capture antibody overnight at 4°C. After washing and blocking with blocking buffer R for 1 hour at 37°C, freshly isolated splenocytes (5 × 10^5^) were added to the wells with or without peptides in medium containing RPMI 1640, 10% FCS, 2 mM glutamine, 1 mM sodium bicarbonate and 100 μg/ml penicillin-streptomycin. Individual peptides or peptide pools were added at 5 μg/ml. For positive controls, PMA (50 ng/ml) and Ionomycin (1 μg/ml) were added to the Elispot wells. Plates were then incubated at 37°C for 24-28 hours. After washing, biotinylated detection antibody was added to wells, and plates were then incubated for 1 hour at 37°C. After further washing, streptavidin-horseradish peroxidase was added for a further 1 hour incubation at 37°C. After washing again, spots were revealed by adding AEC (3-amino-9-ethylcarbazole) substrate solution to yield a coloured spot after a 20-40 minutes incubation at room temperature in the dark. Finally, colour development was stopped by thoroughly rinsing with tap water.

The number of spots was analysed with a fully automated computer-assisted video imaging analysis system (Bioreader 4000, Germany). The average number of spot-forming cells (SFC) was adjusted to 1 × 10^6 ^splenocytes for data display.

#### 2.4.3 Intracellular cytokine staining and flow cytometry analysis

The splenocytes from the mice immunised with virus rVVJ16/18E7E6 and virus rVVJ1175 were cultured in 96 well plates at a concentration of 2 × 10^6 ^cells/well and stimulated with or without 5 μg/ml of HPV16E7_49-57 _peptide or HPV18E6_67-75 _peptide for 5 hours. For the last 2 hours of stimulation, 10 μM monensin was added to block the secretion of cytokines. Cells were then washed and stained for cell surface markers CD8 and CD4. After fixation with 4% paraformaldehyde and permeabilisation with 0.15% saponin, cells were then stained with APC-conjugated anti-IFN-γ antibody. The labelled cells were analysed on a FACS Calibur cytometer.

#### 2.4.4 *In vivo *cytotoxicity assay

The *in vivo *cytotoxicity assay was performed as described by Barber et al. [27]. In brief, splenocytes from naive C57BL/6 mice were labelled with either 1 μM or 10 μM of the cytosolic dye CFSE. The cells labelled with 1 μM CFSE were left unpulsed with peptide, and the cells labelled with 10 μM CFSE were pulsed with 5 μg/ml HPV16E7_49-57 _or HPV18E6_67-75 _peptide for 4 hours. Next, the pulsed and unpulsed cells were mixed in a 1:1 ratio and transferred intravenously (10 million cells for each mouse) into the mice vaccinated with virus rVVJ16/18E7E6 or virus rVVJ1175 or into uninfected mice. After 15 hours of *in vivo *killing, splenocytes were isolated and analysed by flow cytometry to measure target cell clearance. The percentage of specific killing was calculated as follows: 100-[(% peptide-pulsed in infected/% unpulsed in infected)/(% peptide-pulsed in uninfected/% unpulsed in uninfected)] × 100.

#### 2.4.5 *In vivo *tumour protection and treatment experiments using the TC-1 tumour cell line

For *in vivo *tumour protection experiments, C57BL/6 mice (ten animals per group) were immunised twice intraperitoneally at two-week intervals with 1 × 10^7 ^pfu recombinant vaccinia virus rVVJ16/18E7E6, virus rVVJ16E6E7, virus rVVJ1175 or PBS. Two weeks after the last vaccination, mice were challenged subcutaneously in the groin with 1.2 × 10^4 ^TC-1 tumour cells. In the treatment experiments, C57BL/6 mice were challenged with 1 × 10^4 ^TC-1 cells at day 0 and then vaccinated at day 1 and day 11 with the same dose as that used in the tumour protection experiment. Tumour development was monitored twice a week during a 50 day follow-up.

### 2.5 Rhesus monkey studies

#### 2.5.1 Rhesus monkeys and immunisation

Rhesus monkeys aged between 4 and 5 years old and weighing between 2 and 3 kg were used in this study. These monkeys were kept indoors in individual cages with artificial lighting (12 h dark/12 h light cycle) and air-conditioning that maintained the ambient temperature at 21-25°C. All animals were routinely examined by trained veterinarians and cared for in accordance with the approved guidelines.

In this study, a total of 9 rhesus monkeys were used and randomly divided into three groups without regard for gender (three monkeys/per group). The monkeys were immunised twice intradermally in the left leg with PBS (numbers 1, 2 and 3), rVVJ1175 (numbers 4, 5 and 6) or rVVJ16/18E7E6 (numbers 7, 8 and 9). They were primed with 1 × 10^7 ^pfu recombinant vaccinia virus at day 0 and boosted with 5 × 10^7 ^pfu recombinant vaccinia virus at day 11. Peripheral blood mononuclear cells (PBMCs) of all three groups of vaccinated monkeys were analysed for HPV-specific cellular immunity by IFN-γ Elispot assays at days -2 and 53.

#### 2.5.2 Monkey IFN-γ Elispot assay

The monkey IFN-γ Elispot assay was carried out according to the manufacturer's instructions (U-CyTech, Netherlands), which was similar to the protocol for mouse. Briefly, the PBMCs from rhesus monkeys were freshly isolated and added at 3 × 10^5^/well with or without peptide pools (pools for E7 or E6 of HPV16 and HPV18) at 5 μg/ml into a PVDF membrane-bottomed plate pre-coated with anti-monkey IFN-γ capture antibody. The plates were then incubated for 24-28 hours at 37°C prior to the detection of specific T cell responses and then developed as for mouse Elispot assays, except that an anti-monkey detection antibody was added. Finally, the average number of SFC was adjusted to 1 × 10^6 ^PBMC for data display.

### 2.6 **Statistical analyses**

Data are presented as means and standard errors. Statistical analyses were performed with GraphPad Prism version 5.01 (GraphPad Software, Inc., 2007). Comparisons of mean immune responses among groups of mice were performed by analyses of variance with an unpaired t test. Comparisons of the percentage of specific killing were performed with Fisher's exact test, and comparisons of survival curves were performed with the log-rank test. In all cases, P values of less than 0.05 were considered to be significant.

## 3. Results

### 3.1 Construction of recombinant vaccinia virus

The recombinant vaccinia virus rVVJ16/18E7E6 expressing HPV 16 and 18 E7E6 fusion proteins was constructed by homologous recombination in CEF cells with the shuttle plasmid pneoTK16/18E7E6 and the parent virus rVVJ1175. The resulting rVVJ16/18E7E6 was purified and amplified. Figure 1B shows the structures of rVVJ16/18E7E6. The target genes were integrated into the TK region of the J fragment within the vaccinia virus genome. The fused, modified HPV16E7E6 gene under the control of the vaccinia-specific 7.5 K promoter was in a back-to back orientation with the fused, modified HPV18E7E6 gene under the control of the vaccinia-specific H6 promoter.

Two pairs of primers (P7.5/TKR and P7.5R/TKL) with the promoter sequence and the bilateral sequence of recombinant vaccinia virus were used to amplify the target genes and flanking sequences by PCR of the extracted virus DNA. The amplified PCR fragments for the two pairs of primers shown in Figure 1C were 1011 bp and 1197 bp, respectively. The real and predicted sizes for both PCR products appeared to be consistent. The sequencing results of the PCR products confirmed that the E7E6 fusion genes were correct and integrated into the right region within the rVVJ16/18E7E6 genome. No comparable fragments were amplified from the control rVVJ1175 or the blank.

Cells infected with rVVJ16/18E7E6 were analysed by western blot using specific antibodies for the HPV16/18 E7 and E6 proteins. Figure 1D shows that rVVJ16/18E7E6 expressed proteins of the size expected for the HPV16 E7E6 fusion protein (30 kD) and HPV18 E7E6 fusion protein (31 kD), whereas the control rVVJ1175 did not produce these proteins.

Furthermore, the genetic stability of bivalent rVVJ16/18E7E6 was tested after 10 passages in CEF, and the results show that the exogenous gene sequences and fusion protein expression levels were stable and that the rates of gene deletion were from zero to 2% (data not shown).

### 3.2 T cell immune responses against HPV16 E7- and HPV18 E6-derived peptides after immunisation with rVVJ16/18E7E6 in mice

**Figure 2:**
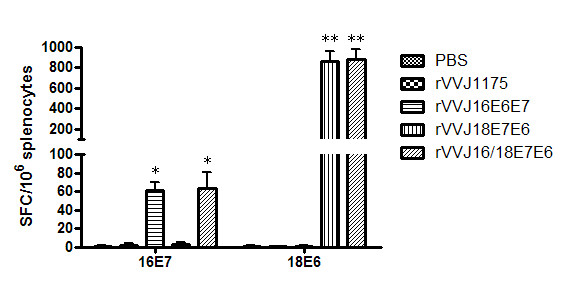
**Specific T cell responses were tested directly *ex vivo *in IFN-γ Elispot assays against peptides HPV16E7_49-57 _and HPV18E6 _67-75 _**. C57BL/6 mice (n = 4 per group) were immunised with PBS, rVVJ1175, rVVJ16E6E7, rVVJ18E7E6 or rVVJ16/18E7E6. Five hundred thousand splenocytes were incubated *ex vivo *with or without 5 μg/ml of HPV16E7_49-75 _or HPV18E6_67-75 _peptides. Plates were incubated for 24-28 hours at 37°C. Background IFN-γ production was generally found for the medium without peptides to be below 5 spots/million splenocytes. Asterisks represent statistically significant differences relative to the rVVJ1175 virus control (* p < 0.05, ** p < 0.001). Data shown are means ± standard deviations for three independent experiments.

To test the specific T cell immune responses induced by recombinant vaccinia virus, C57BL/6 mice were injected with rVVJ16/18E7E6, rVVJ16E6E7, rVVJ18E7E6, rVVJ1175 or PBS. The splenocytes from immunised mice were tested directly *ex vivo *in IFN-γ Elispot assays against peptides HPV16E7_49-57 _and HPV18E6_67-75 _and peptide pools of both HPV16 E6 and HPV18 E7. No significant IFN-γ responses were detected for the HPV16 E6 and HPV18 E7 peptide pools in any of the immunised mice. However, as shown in Figure 2, rVVJ16/18E7E6 was able to induce specific cellular immune responses against peptides HPV16E7_49-57 _and HPV18E6_67-75 _in C57BL/6 mice after two immunisations, and the average numbers of IFN-γ-positive spots were 63.6 ± 16 and 881.4 ± 96 SFC/10^6 ^splenocytes. The immune responses were significantly increased when compared with the control mice immunised with rVVJ1175 or PBS (P < 0.05 and P < 0.001, respectively), and they were similar to the immune responses in mice immunised with virus rVVJ16E6E7 (HPV16E7_49-57_, 61.0 ± 8.7) and with virus rVVJ18E7E6 (HPV18E6_67-75_, 858.4 ± 98).

**Figure 3:**
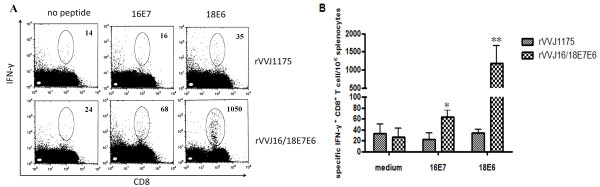
**Intracellular cytokine staining with flow cytometry analysis to determine the proportion of specific IFN-γ -expressing CD8^+ ^T cells**. Splenocytes from mice (n = 3 per group) immunised with virus rVVJ16/18E7E6 and virus rVVJ1175 were cultured and stimulated with either HPV16E7_49-57 _or HPV18E6_67-75 _peptide. Splenocytes without peptide stimulation were used as a negative control. The splenocytes were stained for both CD8^+ ^and intracellular IFN-γ (A) Representative intracellular cytokine staining. The number of CD8^+ ^IFN-γ ^+ ^T cells in 1 × 10^6 ^splenocytes are indicated in the upper right corner. (B) Bar graph depicting the number of specific IFN-γ expressing CD8^+ ^T cells per 1 × 10^6 ^splenocytes (mean ± SD) following *in vitro *stimulation in two independent experiments. Asterisks represent statistically significant differences relative to the rVVJ1175 virus control (* p < 0.05, ** p < 0.001).

To determine whether the IFN-γ production was induced by CD8^+ ^T cells, intracellular cytokine staining was performed. Splenocytes from mice immunised with virus rVVJ16/18E7E6 and virus rVVJ1175 were stimulated *in vitro *with peptides HPV16E7_49-57 _and HPV18E6_67-75_. As shown in Figures 3A and 3B, the frequency of specific IFN-γ producing CD8^+ ^T cells were 63.4 ± 12 and 1188 ± 485 per 10^6 ^splenocytes in the mice immunised with virus rVVJ16/18E7E6; these frequencies were significantly increased compared with mice immunised with rVVJ1175 (22 ± 11 (P < 0.05) and 34.5 ± 6 (P < 0.001), respectively). Specific IFN-γ producing CD4^+ ^T cells were not detected in any group of immunised mice (data not shown).

**Figure 4:**
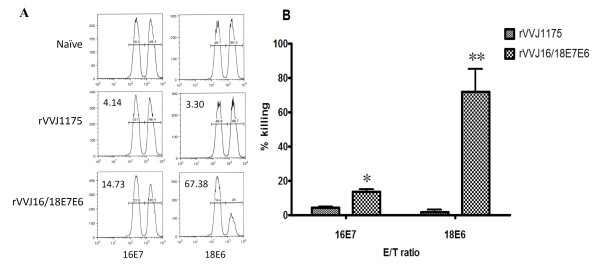
***In vivo *cytotoxicity assays to determine the specific lytic potential of effector CD8^+ ^T cells in mice immunised with rVVJ16/18E7E6**. (A) Target cells were transferred into mice (n = 3 per group) immunised with virus rVVJ16/18E7E6 or virus rVVJ1175 or into naïve mice. Numbers represent the percentage of target cells killed. (B) The bar graph indicates the percentage of specific killing for peptides HPV16E7_49-57_- and HPV18E6_67-75 _-pulsed targets from the indicated groups of mice. Data are given as mean ± SD of two independent experiments. The percentage of specific killing was calculated as follows: 100-[(% peptide pulsed in infected/% unpulsed in infected)/(% peptide pulsed in uninfected/% unpulsed in uninfected)] × 100. Asterisks represent statistically significant differences relative to the rVVJ1175 virus control (* p < 0.05, ** p < 0.001).

In order to assess the lytic potential of effector CD8^+ ^T cells in mice immunised with rVVJ16/18E7E6, we examined killing cells *in vivo *by transferring target cells pulsed with peptides HPV16E7_49-57 _and HPV18E6_67-75 _into immunised mice. As shown in Figure 4A and 4B, 13.56% of the HPV16E7_49-57 _peptide-pulsed targets and 71.96% of the HPV18E6_67-75 _peptide-pulsed targets were eliminated in mice immunised with virus rVVJ16/18E7E6. Only 4.2% and 1.65% targets cells were killed in control mice immunised with rVVJ1175. These differences were statistically significant, and P values were less than 0.05 and 0.001.

Taken together, these results indicated HPV16E7 and HPV18E6 specific cellular immune responses were induced in mice immunised with virus rVVJ16/18E7E6.

### 3.3 *In vivo *tumour protection and treatment experiments using the TC-1 tumour

**Figure 5:**
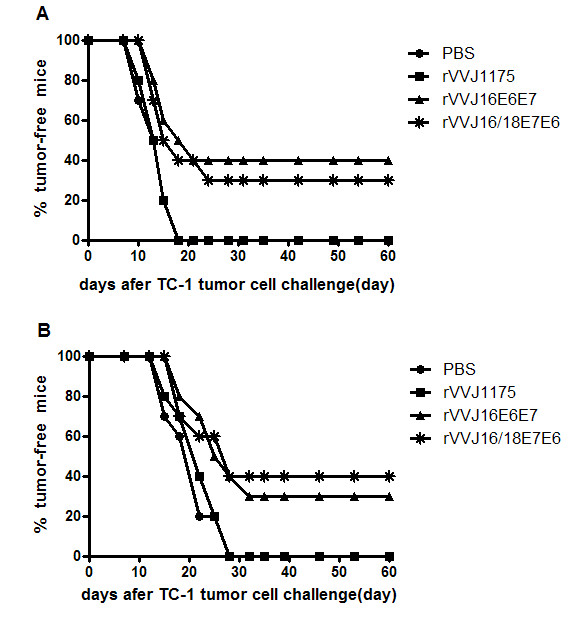
***In vivo *tumour protection and treatment experiments using TC-1 tumour cells with recombinant vaccinia virus**. Panel A: *in vivo *tumour protection experiments; C57BL/6 mice (n = 10 per group) were immunised intraperitoneally twice at two weeks interval with 1 × 10^7 ^pfu recombinant vaccinia virus rVVJ16/18E7E6 and rVVJ16E6E7, control virus rVVJ1175 or PBS. Two weeks after the last vaccination, mice were challenged subcutaneously in the groin with 1.2 × 10^4 ^TC-1 tumour cells. Panel B: the treatment experiments; C57BL/6 mice (n = 10 per group) received 1 × 10^4 ^TC-1 cells on day 0 and were vaccinated with in same groups and dose as used in panel A at days 1 and 11. Tumour development was monitored twice a week during a 50-day follow-up. The data in this figure are from one of two similar experiments.

In order to assess the prophylactic antitumour efficacy of rVVJ16/18E7E6 vaccination in the HPV16^+ ^TC-1 tumour model where T cells specific for the immunodominant D^b ^HPV16E7_49-57 _epitope would participate, C57BL/6 mice were vaccinated twice with rVVJ16/18E7E6, rVVJ16E6E7, rVVJ1175 or PBS at two-week intervals and challenged with 1.2 × 10^4 ^TC-1 tumour cells two weeks after the last vaccination. As shown in Figure 5A, all control mice receiving either rVVJ1175 or PBS developed tumours within 18 days following tumour challenge. However, the mice immunised with rVVJ16/18E7E6 delayed tumour development and were partially protected (30%, P = 0.039) against the challenge with TC-1 tumour cells. Similar results were observed in mice immunised with rVVJ16E6E7, which can provide 40% protection (P = 0.011).

We aimed to construct rVVJ16/18E7E6 to develop a therapeutic vaccine for the treatment of HPV16^+ ^and HPV18^+ ^cancers, so we examined whether the recombinant vaccinia virus could be used in a therapeutic setting. C57BL/6 mice were inoculated with 1 × 10^4 ^TC-1 tumour cells on day 0. Mice were then immunised and boosted with rVVJ16/18E7E6, rVVJ16E6E7, rVVJ1175 or PBS at days 1 and 11 after tumour cell inoculation. As shown in Figure 5B, the results were similar to the prophylactic antitumour efficacy for rVVJ16/18E7E6. Mice in the group treated with rVVJ16/18E7E6 or rVVJ16E6E7 post-TC-1 cell challenge appeared to have delayed tumour development and partial protection (40% or 30%). All control mice receiving either rVVJ1175 or PBS developed tumours within 28 days in this therapeutic setting. These difference were statistically significant compared with the control virus rVVJ1175 or PBS (P < 0.05).

### 3.4 Vaccination with rVVJ16/18E7E6 generates E6- and E7-specific T cell immune responses in rhesus monkeys

As specific T cell immune responses against peptides HPV16E7 and HPV18E6 were induced in immunised mice, we examined whether our recombinant vaccinia virus could elicit anti-tumour cellular immune responses in non-human primates. Therefore, we tested the immunogenicity of rVVJ16/18E7E6 in rhesus monkeys. The monkeys were immunised twice with the virus at days 0 (1 × 10^7 ^pfu) and 11 (5 × 10^7 ^pfu). Control monkeys received rVVJ1175 or PBS. PBMCs from the monkeys were analysed for HPV-specific cellular immunity against peptide pools from E7 or E6 of HPV16 and HPV18 by IFN-γ Elispot assays at days -2 and 53.

**Figure 6:**
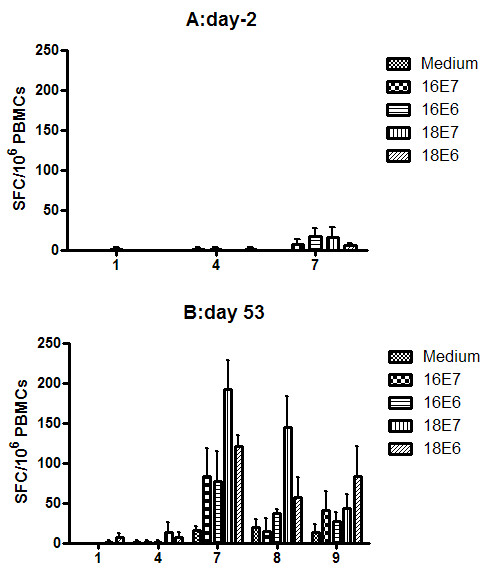
**Specific T cell responses were tested directly *ex vivo *in IFN-γ Elispot assays with PBMCs in rhesus monkeys**. The rhesus monkeys (n = 3 per group) were immunised intradermally in the left leg twice with PBS (numbers 1, 2 and 3), rVVJ1175 (numbers 4, 5 and 6) or rVVJ16/18E7E6 (numbers 7, 8 and 9). The monkeys were primed with 1 × 10^7 ^pfu recombinant vaccinia virus at day 0 and boosted with 5 × 10^7 ^pfu recombinant vaccinia virus at day 11. PBMCs from all three groups of vaccinated monkeys were analysed for HPV-specific cellular immunity against medium (without peptides) and peptide pools E7 or E6 of HPV type 16 and 18 by IFN-γ Elispot assays at days -2 and 53.

There were no detectable T cellular immune responses specific for HPV peptide pools in any of the monkeys on days -2. Figure 6A shows the IFN-γ responses on day -2 for three individual monkeys (numbers 1, 4 and 7) from each of the three groups, which represents the background immune response (< 20 SFC/10^6 ^PBMCs).

However, there were significant specific immune responses detected in the monkeys immunised with rVVJ16/18E7E6 at day 53. As shown in Figure 6B, the responses for cells stimulated with medium without peptides ranged from 0 to 17 SFC/10^6 ^PBMCs. Specific T cell immune responses against the four peptide pools were induced in the rhesus monkeys vaccinated with rVVJ16/18E7E6, among which responses to peptide pools HPV16 E7 and E6 were detected in one monkey (number 7), and the average number of IFN-γ spots were 83 ± 35 and 77 ± 38 SFC/10^6 ^PBMCs. Responses to peptide pool HPV18 E7 occurred in two monkeys (numbers 7 and 8) and the spots were 192 ± 37 and 144 ± 39 SFC/10^6 ^PBMCs. Responses to peptide pool HPV18 E6 were observed in two monkeys (numbers 7 and 9), and spots were 121 ± 14 and 83 ± 38 SFC/10^6 ^PBMCs. Among the three monkeys generating specific responses, one (number 7) responded to all 4 peptide pools. In contrast, no specific responses were detected in control monkeys injected with rVVJ1175 or PBS. Hence, immunisation of rhesus monkeys with rVVJ16/18E7E6 was able to elicit E7 and E6 specific cellular responses for HPV16 and HPV18, respectively.

## 4. Discussion

Persistent infection with high-risk human papillomavirus (HPV) is a predominant cause of cervical cancer, and HPV16 and HPV18 occur in 50% and 20% of cervical cancer cases, respectively[3]. Therefore, the development of therapeutic vaccines is important for the treatment of HPV-associated neoplasms, such as cervical cancer and its intraepithelial precursor lesions. In this study, we constructed a recombinant vaccinia virus (rVVJ16/18E7E6) co-expressing the HPV16/18 E7E6 fusion proteins as a candidate therapeutic vaccine to protect against both HPV16 and HPV18. For safety future clinical application, we modified the E6 and E7 genes to inactivate their oncogenic activity and fused them in one reading frame.

Because cell-mediated immunity appears to be important in controlling HPV infections and disease, we selected a vaccinia virus of the Tiantan strain to construct the recombinant vaccinia virus rVVJ16/18E7E6 for the delivery of the HPV gene sequences. We expected that the recombinant virus would be able to induce a strong cellular immune response to control or eliminate HPV-infected cells and thus treat the HPV-associated neoplasms. In this study, we showed that the recombinant virus encoded the HPV DNA sequences in the expected configuration and that the heterologous coding sequences were successfully expressed as protein products in the virus-infected cells. We also showed that a specific T cell response to peptide HPV16 E7_49-57 _could be elicited in the C57BL/6 mice immunised with the recombinant vaccinia virus rVVJ16/18E7E6 using two in vitro assays. Furthermore, we also showed that specific lytic activity could be detected in immunised mice *in vivo *and resulted in antitumour responses in HPV16^+ ^TC-1 tumour protection and treatment experiments, including delayed tumour appearance and 30-40% protection. These results were similar to the immune responses to rVVJ16E6E7 (one recombinant vaccinia virus expressing HPV16 E6E7 fusion protein). These data suggest that peptide HPV16 E7_49-57_, described by other studies as the immunodominant epitope associated with MHC class I of the H-2^b ^haplotype, could be efficiently presented in C57BL/6 mice immunised with rVVJ16/18E7E6.

Vaccination with virus rVVJ16/18E7E6 in C57BL/6 mice was able to induce significant CD8^+ ^T cell responses against peptide HPV18E6_67-75_, as shown by *in vitro *or *in vivo *assays, which indicated that the HPV18E7E6 fusion protein was effectively processed in C57BL/6 mice. This was unexpected because McCarthy's studies indicated that peptide HPV18E6_67-75 _was only able to bind to HLA-A2 molecules in HLA-A2/K^b ^mice immunised with a HPV18E6 DNA vaccine [28]. Therefore, we hypothesized that this peptide may be presented in humans as well as in the C57BL/6 mice. Although peptide HPV18E6_67-75 _was different from two other reported peptides (the dominant epitope was E6_50-64 _and the subdominant epitope was E6_57-71_) described by mapping HPV18E6 peptide pools in C57BL/6 mice immunised with a HPV18E6E7 DNA vaccine carried out by Yan and colleagues [29], this peptide overlapped with the peptide E6_57-71 _and was adjacent to the peptide E6_50-64_. This difference may be explained by the fact that the different gene fusion procedure used in our study may have altered antigen presentation of HPV18E6.

We did not detect T cell responses against the HPV16E6 and HPV18E7 antigens. We hypothesize that these epitopes may not be effectively processed due to either the E7E6 gene fusion method or to the fact that they elicited T cell responses that were too low to be detected. Importantly, although there is no animal challenge model available for HPV18, rhesus monkeys immunised with rVVJ16/18E7E6 could elicit E7- and E6-specific cellular responses to both HPV16 and HPV18. In addition, when monkeys were boosted with one protein-based vaccine HPV16L2E7E6 that was expressed efficiently in a prokaryotic system and purified by using ion exchange chromatography, the responses to the HPV16 E7 and E6 peptide pools were able to be improved in two monkeys.

In conclusion, we observed that the recombinant vaccinia virus rVVJ16/18E7E6 was able to generate significant and functional cellular immunity in both mice and rhesus monkeys. These data provide a basis to proceed with the recombinant virus as a potential vaccine candidate in further studies.

## Abbreviations

HPV: human papillomavirus; Elispot: enzyme-linked immunospot assay; CEF: chicken embryonic fibroblasts; AA: amino acid; PCR: polymerase chain reaction; MOI: multiplicity of infection; SFC: spot-forming cells; PBMCs: peripheral blood mononuclear cells; TK: thymidine kinase.

## Competing interests

The authors declare that they have no competing interests.

## Authors' contributions

LZ generated viral constructs, performed immunogenicity studies in mice and rhesus monkeys, and drafted the manuscript. BL contributed ideas to this work and edited the manuscript. JR, JF, and ZP participated in performing the immunogenicity studies in mice and rhesus monkeys. JG and HZ participated in generating the viral constructs and their detection. HT and LR directed the study, analysed and interpreted the data. WT participated in designing the study. All authors read and approved the manuscript.
